# Centrosome dysfunction associated with somatic expression of the synaptonemal complex protein TEX12

**DOI:** 10.1038/s42003-021-02887-4

**Published:** 2021-12-08

**Authors:** Sumit Sandhu, Ieng F. Sou, Jill E. Hunter, Lucy Salmon, Caroline L. Wilson, Neil D. Perkins, Neil Hunter, Owen R. Davies, Urszula L. McClurg

**Affiliations:** 1 Howard Hughes Medical Institute, Department of Microbiology and Molecular Genetics, University of California, Davis, CA 95616 USA; 2 Institute of Systems, Molecular and Integrative Biology, University of Liverpool, Liverpool, L69 7ZB UK; 3 Biosciences Institute, Newcastle University, Newcastle upon Tyne, NE2 4HH UK; 4 Wellcome Centre for Cell Biology, Institute of Cell Biology, University of Edinburgh, Michael Swann Building, Max Born Crescent, Edinburgh, EH9 3BF UK

**Keywords:** Meiosis, Cell growth

## Abstract

The synaptonemal complex (SC) is a supramolecular protein scaffold that mediates chromosome synapsis and facilitates crossing over during meiosis. In mammals, SC proteins are generally assumed to have no other function. Here, we show that SC protein TEX12 also localises to centrosomes during meiosis independently of chromosome synapsis. In somatic cells, ectopically expressed TEX12 similarly localises to centrosomes, where it is associated with centrosome amplification, a pathology correlated with cancer development. Indeed, TEX12 is identified as a cancer-testis antigen and proliferation of some cancer cells is TEX12-dependent. Moreover, somatic expression of TEX12 is aberrantly activated via retinoic acid signalling, which is commonly disregulated in cancer. Structure-function analysis reveals that phosphorylation of TEX12 on tyrosine 48 is important for centrosome amplification but not for recruitment of TEX12 to centrosomes. We conclude that TEX12 normally localises to meiotic centrosomes, but its misexpression in somatic cells can contribute to pathological amplification and dysfunction of centrosomes in cancers.

## Abstract (web summary)

Sandhu et al. report that the synaptonemal complex (SC) protein, TEX12, localises to centrosomes independently of the SC during meiosis. They also show that it provokes centrosome amplification in somatic cells, a pathology associated with cancer development.

## Introduction

Maintaining genome integrity is critical for the healthy growth of somatic cells; consequently, genome instability is one of the hallmarks of cancer^1^. Genome stability must also be maintained through the germline as gametes are formed via meiosis. Meiotic cell division relies on highly complex and synchronised chromosome movements to achieve homologue pairing and crossing-over. Homologous chromosome pairs must become linked by crossovers in order to biorient on the meiosis-I spindle, adding an additional level of complexity relative to mitotic chromosome congression. This complexity is reflected in a dedicated meiotic structure called the synaptonemal complex (SC). This supramolecular protein structure binds together homologous chromosome pairs and is essential for the maturation of inter-homologue crossover recombination events, and thereby fertility^2–6^. Consequently, meiotic proteins responsible for this process are highly specialised and otherwise silenced in somatic cells. However, SC proteins are aberrantly re-expressed in some cancers defining a subset of the so-called cancer-testis antigens^7^. On the basis of their roles in chromosome structure, and their predicted functional interaction with the DNA recombination machinery, we reasoned that SC components could be detrimental to chromosomal stability if expressed outside of the regulated process of meiosis, and thus could contribute to the aetiology and progression of cancer. This study details our investigation into the biology of one of these proteins, namely TEX12.

Here we show that mammalian SC protein TEX12, previously identified as a component of the SC central element, localises to centrosomes during meiosis in males, and upon ectopic expression in somatic cells. TEX12 can localise to centrosomes independently of other SC proteins, and its misexpression in somatic cells is associated with centrosome amplification through a mechanism that involves phosphorylation of a single conserved tyrosine, Y48. Centrosome amplification is reported to be sufficient for oncogenesis^8–10^, although this premise has been contradicted^11–13^, suggesting that oncogenesis induced by centrosome amplification might be context-dependent. However, it is widely accepted that centrosome amplification is associated with cancer progression and poorer prognosis^14–16^. In this manuscript we demonstrate that aberrant expression of TEX12 is common in cancer where it is associated with centrosome amplification and poor prognosis. We further find that stimulation of retinoic acid receptor signalling is required for ectopic expression of TEX12 in somatic cells. Thus, we propose a previously unappreciated localisation for TEX12 and uncover the drivers of its aberrant expression during tumorigenesis.

## Results

### TEX12 is aberrantly expressed in cancer cells

**Fig. 1:**
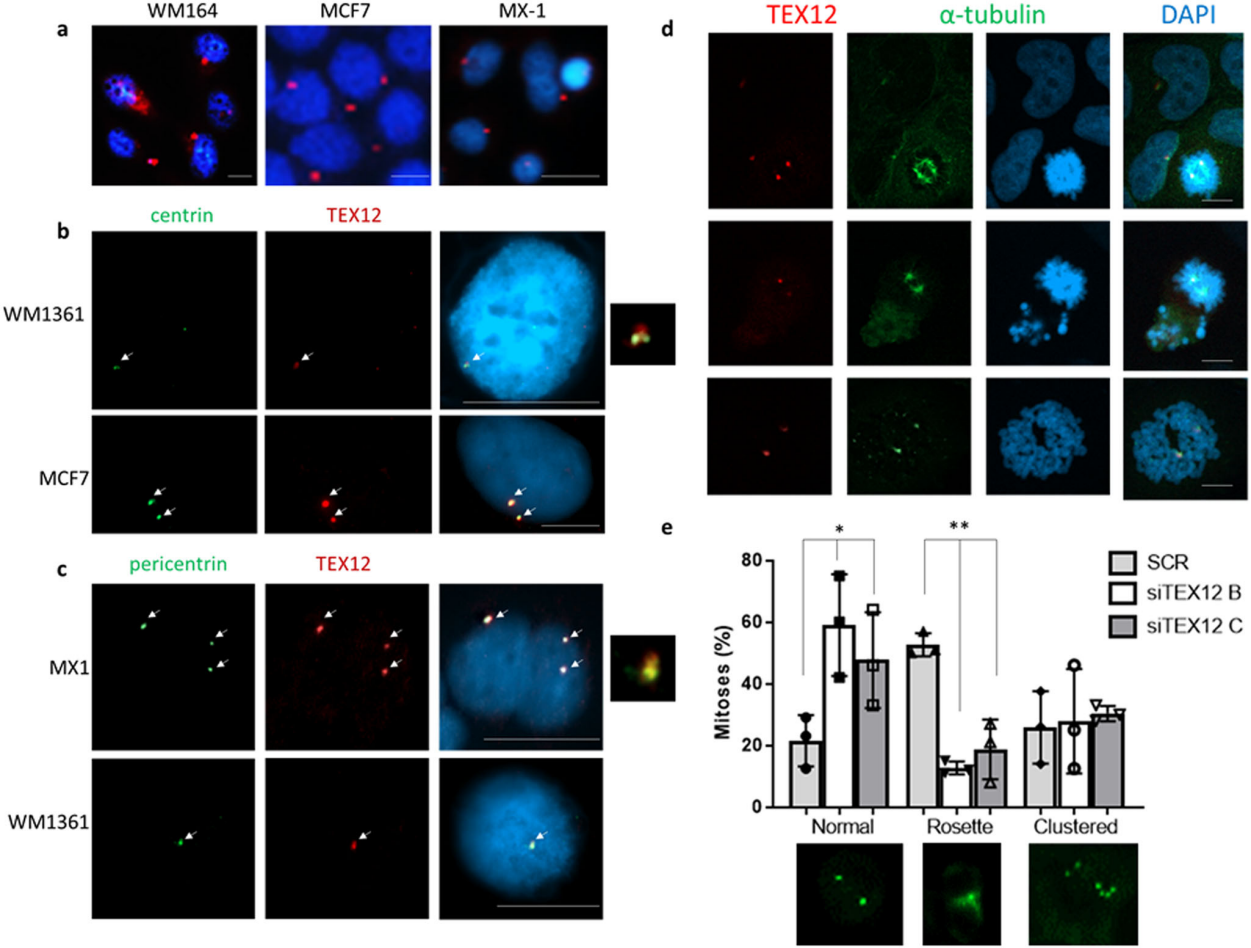
TEX12 is a centrosomal protein in cancer. **a** Cells were stained for TEX12 visible in red with DAPI visible in blue. Bar = 20 µm. **b** Cells were stained for TEX12 (red), centrin (green) and nuclei were visualised in blue with DAPI. Arrows indicate centrosomes. Bar = 20 µm. **c** Cells were stained for TEX12 (red), pericentrin (green) and nuclei were visualised in blue with DAPI. Arrows indicate centrosomes. Bar = 20 µm. **d** Dividing PC3 cells stained for TEX12 visible in red, α-tubulin visible in green and DAPI visible in blue. Bar = 20 µm. **e** Cancer cells were treated with non silencing control (CTR) siRNA, siTEX12 B or siTEX12 C for 96 h followed by centrin staining in green. Mitosis types were quantified in 200 cells from three independent experiments. *p < 0.05; ***p < 0.005. Error bars represent standard deviation.

Meiotic machinery can be harnessed by cancer cells to promote oncogenesis^17–21^. We analysed large-scale transcriptomic datasets of patient samples and observed that a synaptonemal complex protein, TEX12, was aberrantly expressed in a wide variety of tumours in ~10% of patients (ranging between 5 and 17%) (Suppl Fig. 1a). Immunofluorescent staining, performed in a number of cancer cell lines and upon ectopic expression in mitotic cells, consistently revealed expression of TEX12 in dot-like peri-nuclear foci (Fig. 1a and Suppl Fig. 2a, b). The number and location of TEX12 foci resembled that of centrosomes. Accordingly, TEX12 co-localised with pericentrin foci in cancer cells (Fig. 1b, c), confirming its recruitment to the pericentrosomal area of centrosomes furthermore, we have observed centrosomal TEX12 in mitosis (Fig. 1d)

Analysis of TEX12 expressing cancer cell lines revealed high levels of rosette mitoses defined by presence of a centriole rosette at one or both centrosomes^22^, a centrosome phenotype commonly associated with cancer^22^. Rosettes were substantially reduced upon *TEX12* silencing by siRNA (Fig. 1e), implying a cause-and-effect relationship. We therefore set out to investigate if, in keeping with the mechanisms of other meiosis-specific genes in cancer^17,21^, the centrosomal role of TEX12 in cancer cells may represent a pseudo-meiotic function.

### TEX12 assembles into toroidal structures at spermatocyte centrosomes

**Fig. 2:**
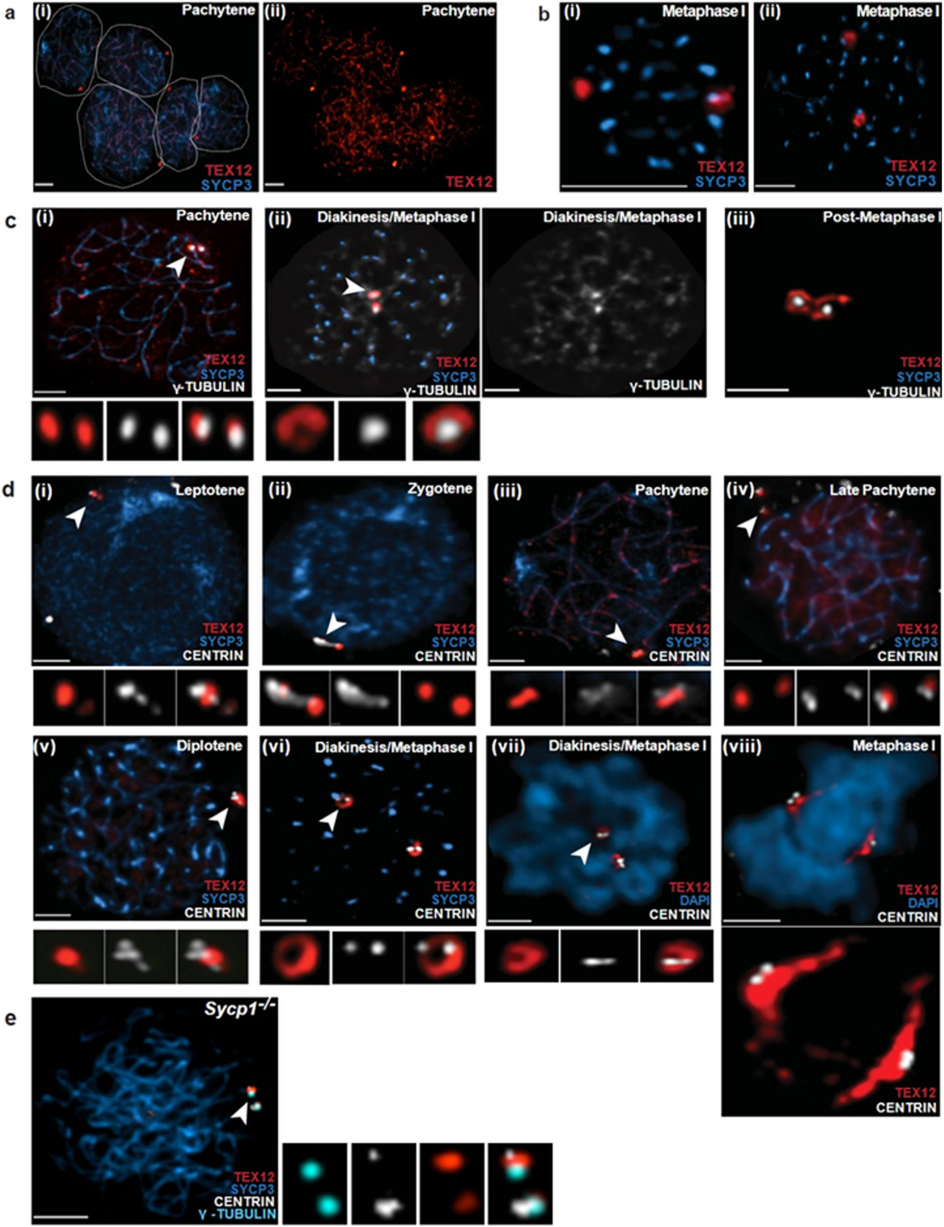
TEX12 localises to centrosomes in mouse meiosis. **a** Pachytene-stage mouse spermatocyte immuno-stained for chromosome axis marker SYCP3 and TEX12. Individual nuclei are outlined. **b** Two views of metaphase-I nuclei with SYCP3 and TEX12 staining. **c** Spermatocyte nuclei at pachytene (**i**), diakinesis/metaphase-I (**ii**) and post-metaphase-I (**iii**), immuno-stained for SYCP3 (blue), TEX12 (red) and γ-Tubulin (white). Centrosomes are highlighted with arrowheads and magnified in the lower panels. **d** Mouse spermatocytes immuno-stained for SYCP3 (blue), TEX12 (red), and centrosomal marker Centrin (white) (**i**–**vi**) or TEX12, Centrin (white) and DAPI (blue) (**vii**–**viii**). Individual centrosomes are highlighted with arrowheads and magnified in the lower panels. For panel (**viii**), both TEX12-staining structures are magnified below. **e** Spermatocyte nuclei from an Sycp1 mutant, immuno-stained for TEX12, Centrin, γ-Tubulin and SYCP3. Centrosome are highlighted with arrowhead and magnified on side. All images are from squash preparations of mouse spermatocyte nuclei. Scale bar in all images, 10 µm.

The localisation of TEX12 to centrosomes in cancer cells raises the possibility that its physiological function during meiosis might also include a role at microtubule organising centres (MTOCs) in addition to its well-established role in chromosome synapsis. Consistently, in mouse meiotic spermatocytes in early prophase I, TEX12 localised to peri-nuclear dot-like foci as well as SCs (Fig. 2a and Suppl Fig. 2a). In metaphase-I nuclei, after the SC disassembles, two prominent TEX12 structures were present that were no longer restricted to a peripheral location (Fig. 2b). To address the possibility that these foci might be centrosome-related structures, spermatocytes were co-stained for TEX12 and the MTOC marker, γ-tubulin (Fig. 2c). In prophase-I spermatocytes, two TEX12 foci partially overlapped with bright γ-tubulin signals (Fig. 2c). During diakinesis/metaphase-I, TEX12 toroids appeared to encircle the two brightly staining γ-tubulin structures (Fig. 2c, ii). Numerous additional γ-tubulin structures appeared at this time, many of which localised to centromere regions (that retained residual SYCP3 protein) and presumably nucleate kinetochore-associated microtubules. In post MI spermatocytes (that had lost residual SYCP3), two bright γ-tubulin-staining MTOCs remained, which were closely apposed and surrounded by interconnected TEX12 toroids (Fig. 2c, iii). We hypothesise that these structures are the precursors of MII spindles.

To confirm that TEX12-stained structures were centrosomes, we immuno-stained mouse spermatocytes for TEX12 and the core centriole marker Centrin (Fig. 2d). Close juxtaposition of TEX12 and Centrin signals was observed throughout meiotic prophase I and metaphase I. In prophase I, TEX12 was localised peripheral to nuclei as an elongated or bi-lobed structure, two closely apposed foci, or two more widely separated foci (Fig. 2d, i–v). Centrin was localised immediately adjacent to these TEX12 structures, typically appearing as three or four distinct foci, indicating that centrosome-like MTOCs had been duplicated. During diakinesis/metaphase-I, TEX12 structures morphed into two internally located toroids, each associated with two Centrin foci atop these structures (Fig. 2d, v–viii). In some metaphase-I nuclei, the two TEX12-Centrin structures were elongated and positioned on either side of the chromosome mass, with the Centrin foci on the outer, poleward faces (Fig. 2d, viii).

Finally, we showed that TEX12 localises to centrosome-related structures in the *Sycp1* knock-out mutant mouse, which lacks the SC central region (Fig. 2e), indicating that the localisation of TEX12 to meiotic centrosomes, and possibly also its function at these organelles, is independent of its synaptic function. Taken together, our localisation studies are consistent with TEX12 being a meiosis-specific component of the outer pericentriolar material (PCM) where it may augment or replace another PCM factor to facilitate meiotic spindle function.

### Ectopically expressed TEX12 perturbs centrosome homeostasis in somatic cells

**Fig. 3:**
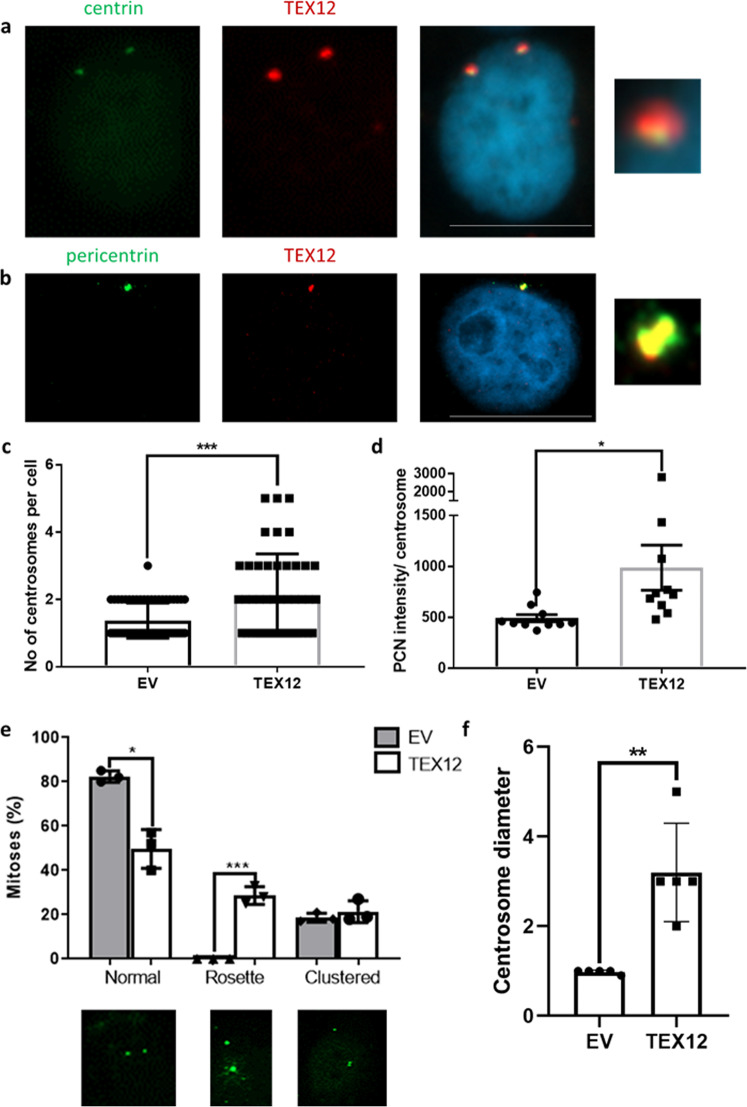
TEX12 regulates centrosome division. **a**, **b** COS-7 cells were transfected with pCMV-HA-TEX12-FLAG for 48 h following by staining for TEX12 (Abcam ab122455) (red) and centrin or pericentrin (green). Bar = 20 µm. **c** Centrosome numbers in COS-7 cells 96 h after transfection with TEX12 (*n* = 45) or empty vector (*n* = 62) were counted in three independent experiments. ***p < 0.005. **d** COS-7 cells were transfected with empty vector control (EV) or TEX12 for 96 h followed by TEX12 and pericentrin imaging. Average pericentrin intensity per centrosome was quantified for 10 cells in each group. *p < 0.05. **e** COS-7 cells were transfected with either empty vector or TEX12 for 96 h followed by centrin staining in green. Mitosis types were quantified in 100 cells from three independent experiments. *p < 0.05; ***p < 0.005. **f** Centrosome size (µm) in COS-7 cells 96 h after transfection with TEX12 or empty vector was quantified in 10 cells in four independent experiments. **p < 0.01. **c**–**f** error bars represent standard deviation.

To confirm that TEX12 can incorporate into centrosomes independently of other SC proteins, we ectopically expressed TEX12 in somatic cells. FLAG-tagged TEX12 expressed in COS7 cells, which do not natively express TEX12 (Suppl Fig. 2a, d), localised to the centrosomes, based on co-staining with centrin (Fig. 3a) and pericentrin (Fig. 3b). Furthermore, TEX12 localisation patterns in somatic cells recapitulated the localisation observed in meiotic cells, with TEX12 colocalizing with pericentrin in dot-like foci (Fig. 3b). Thus, we conclude that TEX12 is recruited to centrosomes in both meiotic and somatic cells independently of other SC proteins.

Cancers are commonly characterised by centrosome amplification and rosette centrosomes that lead to changes in spindle morphology, asymmetric cell division and genome instability^9,10,15,16,22–25^. Thus, given our finding that TEX12 localises to centrosomes, we wondered whether aberrant expression of TEX12 in cancer cells might promote centrosome amplification. Consistently, we observed that centrosome number, determined by counting centrin-staining structures, increased upon TEX12 overexpression (Fig. 3c), with 33% of cells that were transfected with TEX12 (15/45) presenting with more than 2 centrin-staining structures compared to one cell in the vector only control. Moreover, the intensity of pericentrin staining per centrosome was significantly increased (Fig. 3d) and centrosomes appeared larger than those of control cells with the empty vector as assessed by measuring the diameters of centrin-staining structures (Fig. 3f). Notably, we observed that TEX12 expression induced a pattern of enlarged and diffuse centrosomal staining indicative of rosette centrosomes/amplified centrosomes (Fig. 3e), which have been described as a common feature of cancer cells^16,22,26^. Thus, TEX12 is recruited to somatic centrosomes and promotes alterations in centrosome number and morphology. However as TEX12 is not the sole driver of centrosome amplification, silencing its expression in cancer cells characterised by amplified centrosomes was not sufficient to restore normal centrosome numbers even though a non-significant decreasing trend can be observed (Suppl Fig. 1h).

### TEX12 forms a stable homodimer in the absence of SC partners

**Fig. 4:**
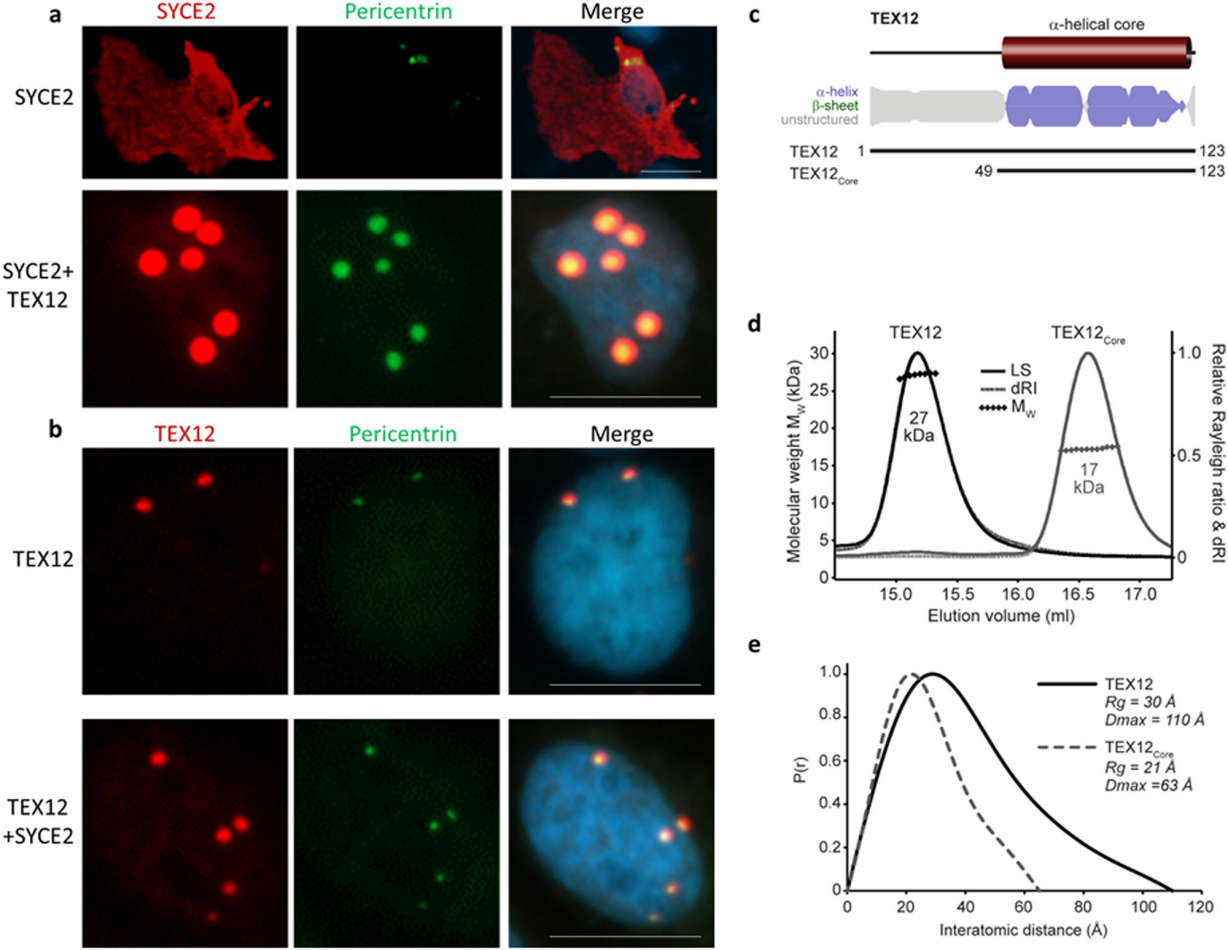
TEX12 forms a stable α-helical dimer and can localise to centrosomes independently of its SC partner, SYCE2. **a** COS-7 cells were transfected with SYCE2 and TEX12 as indicated. Cells were stained for SYCE2 (red) and pericentrin (green). Nuclei were visualised in blue with DAPI. Bar = 20 µm. **b** COS-7 cells were transfected with SYCE2 and TEX12 as indicated. Cells were stained for TEX12 (red) and pericentrin (green). Nuclei were visualised in blue with DAPI. Bar = 20 µm. **c** Sequence analysis of human TEX12 demonstrating the presence of a core α-helical region in the C-terminal half of the protein. Secondary structure prediction is shown as per residue scores and protein constructs are indicated along with their sequence boundaries. **d** SEC-MALS analysis of TEX12 and TEX12core in which light scattering (LS) and differential refractive index (dRI) are overlaid, with and fitted molecular weights (Mw) plotted as diamonds across elution peaks. TEX12 and TEX12core are both dimeric, with molecular weights of 27 kDa and 17 kDa, respectively (theoretical dimer sizes − 28 kDa and 18 kDa). **e** SEC-SAXS analysis of TEX12 and TEX12core. P(r) distributions of TEX12 (solid line) and TEX12core (dashed line) showing maximum dimensions of 110 Å and 63 Å, respectively.

What is the structural basis of the centrosomal recruitment of TEX12? Within the SC, 2:2 and 4:4 SYCE2:TEX12 complexes undergo self-assembly into long fibres that are thought to enable SC growth along the entire length of meiotic chromosomes^27–30^. However, SYCE2 was not required for centrosomal localisation of TEX12 (Fig. 4a, b), raising the question of whether SYCE2-binding is compatible with TEX12 recruitment to centrosomes. The isolated expression of SYCE2 in COS7 cells produced a predominantly cytoplasmic staining pattern (Fig. 4a). However, co-expression of SYCE2 and TEX12 led to SYCE2 recruitment to centrosomes (Fig. 4a, b). Thus, whilst TEX12 is recruited to centrosomes in the absence of SYCE2, centrosome-associated TEX12 retains its ability to bind SYCE2.

In agreement with an SYCE2-independent localisation of TEX12 at centrosomes, we found that recombinant human TEX12 was soluble and stable in isolation (Fig. 4c and Supplementary Fig. 3a, b). Analysis by Circular Dichroism (CD) and Size Exclusion Chromatography Multi-Angle Light Scattering (SEC-MALS) revealed that TEX12 is a largely α-helical homodimer, with a melting temperature of approximately 60° C (Fig. 4d and Supplementary Fig. 3c, d). Further, SEC-MALS and Small-Angle X-ray Scattering (SAXS) determined that the dimeric structural core is provided by amino-acids 49–123 (Fig. 4d, e and Supplementary Fig. 3e, f), corresponding to the same region that interacts with SYCE2^30^. Thus, TEX12 can adopt alternative conformations of an isolated homodimer or within 2:2, 4:4 and fibrous SYCE2:TEX12 complexes, through the same α-helical core.

### A conserved sequence motif in TEX12 is important for the centrosome amplification

**Fig. 5:**
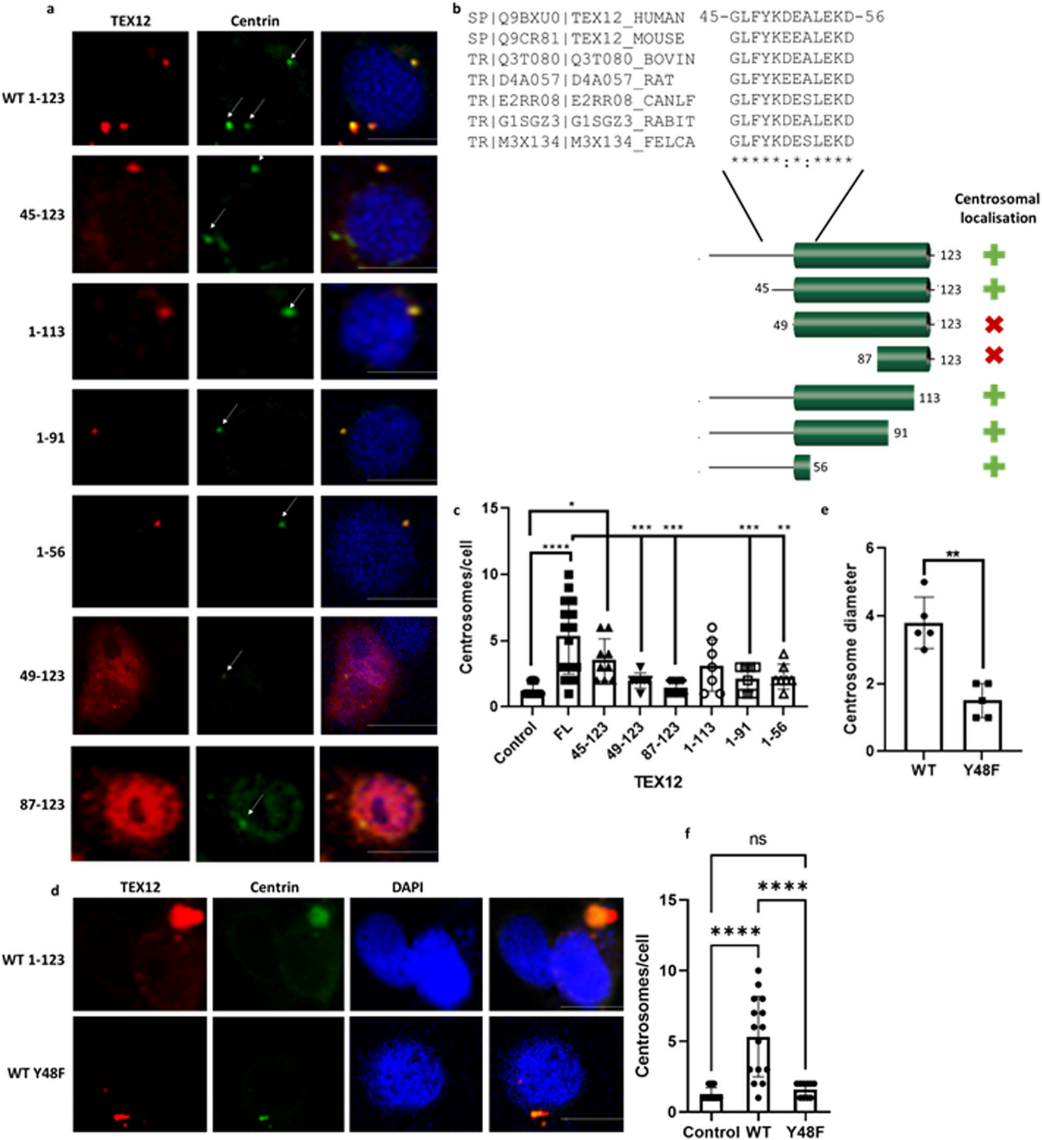
G45-D56 region of TEX12 regulates centrosomal localisation and function. **a** COS-7 cells transfected with TEX12 plasmids as indicated and stained with TEX12 (Abcam ab122455) (red) and centrin (green). Centrosomes are indicated with white arrows. Bar = 20 µm. **b** Diagram of TEX12 domain structure and comparison of region conservation across species. **c** Number of centrosomes per cell in COS-7 cells following transfection with indicated TEX12 plasmids. *p < 0.05; **p < 0.001; ***p < 0.005. Error bars represent standard deviation. Each data point represents an individual cell, between 5 and 15 cells per transfection were measured over three independent experiments. **d** COS-7 cells were transfected with wild-type (WT) and Y48F TEX12 mutant as indicated. Bar = 20 µm. **e** Centrosome size in COS-7 cells 96 h after transfection with WT TEX12 (*n* = 5) or Y48F TEX12 (*n* = 5) was quantified. **p < 0.01. Error bars represent standard deviation across three independent experiments. **f** Number of centrosomes per cell in COS-7 cells following transfection with WT or Y48F TEX12 ****p < 0.001. Error bars represent standard deviation. Each data point represents an individual cell, between 5 and 15 cells per transfection were measured over three independent experiments, this is an expansion of **e**.

To pinpoint the molecular determinants of TEX12 centrosomal recruitment, we generated a series of TEX12 truncation variants based on our biochemical characterisation of its structural and unstructured regions. We found that the structural core (49–123) alone was not recruited to centrosomes at the same rate as full-length protein (Fig. 5a). However, the presence of an additional four amino-acids at the N-terminal end of the structural core (45–123) was sufficient to mediate recruitment comparable to full-length protein. To dissect this further, we analysed C-terminal truncations of the TEX12 structural core that preserve its unstructured N-terminus (Fig. 5a). We observed centrosomal localisation for all C-terminal truncations (expressed proteins correspond to amino-acid residues 1–113, 1–91 and 1–56) (Fig. 5a). By contrast, and similar to truncation 49–123, centrosomal localisation was diminished for TEX12 87–123 and localisation outside of centrosomes was observed for both constructs. Thus, we conclude that the ability of TEX12 to efficiently localise to centrosomes is dependent on the presence of an evolutionarily conserved four amino-acid sequence motif, 45-GLFY-48 (Fig. 5b), that is not required for its structural stability as a homodimer (Fig. 4). Whilst full-length TEX12 led to significant centrosome over-amplification, this effect was not fully replicated by truncation variants that retained centrosome recruitment, indicating that the full sequence is required for TEX12 functionality at centrosomes (Fig. 5c). To pinpoint its molecular mechanism of action, we investigated the role of a highly conserved Y48 residue (Fig. 5b). Mining of public mass-spectrometry datasets identified TEX12 peptides containing Y48 in the phosphorylated form from human lymphoma cell lines (Phosphosite; dataset 12495), suggesting that this might be a functional phosphorylation site. We applied kinase prediction algorithms to the G45-D56 TEX12 peptide, which highlighted seven kinases with the predicted ability to phosphorylate this site, namely CSK, DDR, EGFR, SRC, SYK, EPH and TEC, several of which are known oncogenes^31–33^. Furthermore, all of these kinases, apart from TEC, are known to play a role in meiosis^34–38^. For these reasons, we generated a Y48F TEX12 phospho-null mutant to determine the role of this conserved phosphorylation site on the ability of TEX12 to promote centrosome amplification and increase centrosome size. Whilst the TEX12-Y48F mutant protein was recruited to centrosomes, it had no effect on centrosome number or size (Fig. 5d–f). Thus, the Y48 phosphorylation site is important for the centrosomal pathology in TEX12 positive cancer cells.

### TEX12 expression is an early event in tumourigenesis

**Fig. 6:**
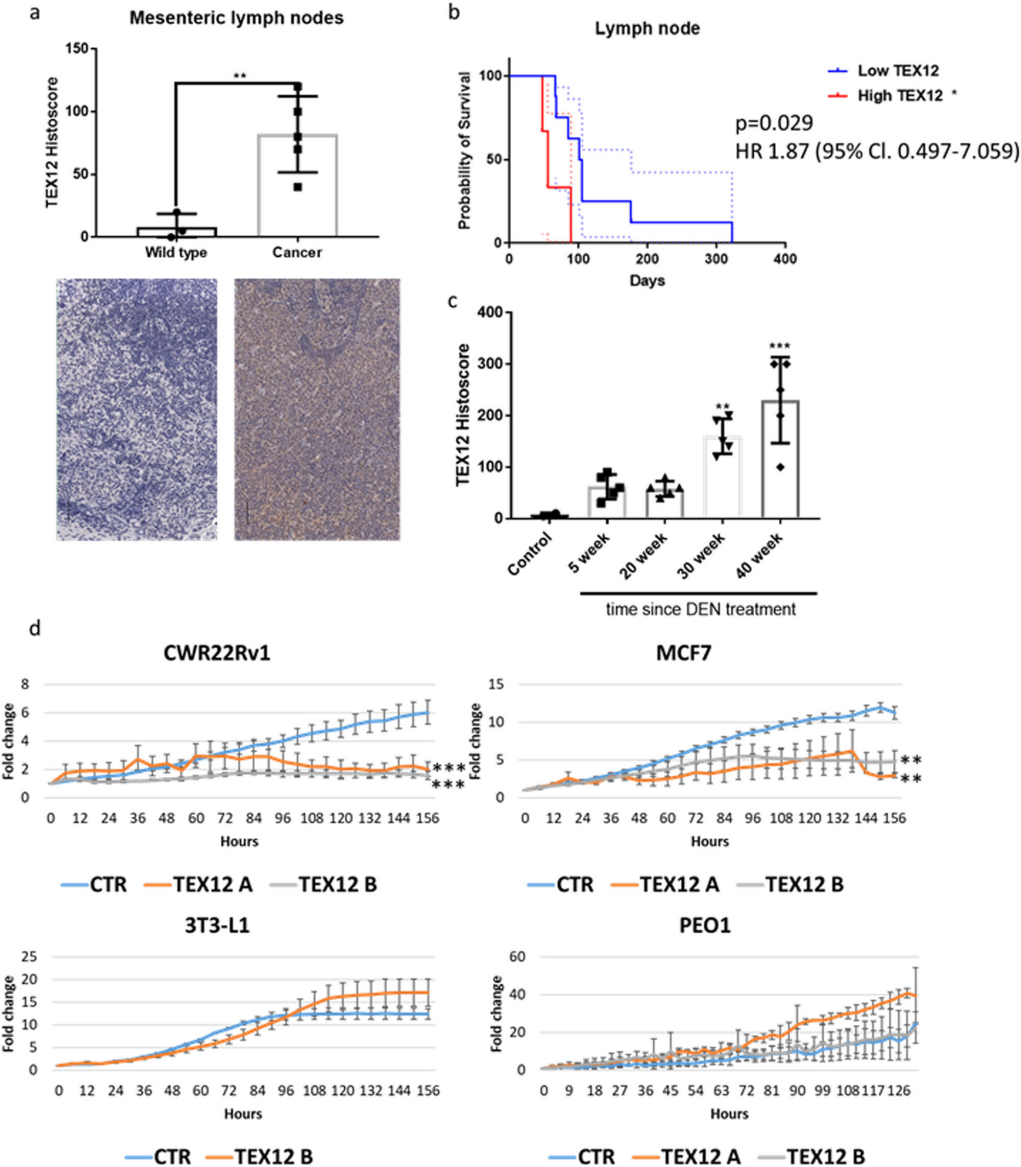
TEX12 is expressed with the onset of cancer and is required for cancer cell proliferation. **a** Eµ-Myc lymphoma and healthy controls mesenteric lymph nodes were stained and scored for TEX12 protein levels. Each data point indicates one animal (*n* = 11). Black lines indicate 50 µm. **p < 0.01. Error bars represent standard deviation. **b** Kaplan–Meier survival curve of Eµ-myc lymphoma mice divided based on their lymph node TEX12 protein histoscore. (*n* = 11). 95% confidence intervals are indicated with dotted lines. **c** Mice have been scored for liver TEX12 protein levels following DEN-treatment tumour induction. Each data point represents one animal (*n* = 22). Error bars represent standard deviation. **d** Cells were treated with control siRNA (CTR) or two TEX12 targeting sequences A or B (details in Methods section) and cell growth was measured in three independent experiments every 3 h with IncuCyte. *p < 0.05; **p < 0.001; ***p < 0.005. Data are presented ±SEM.

To validate our inference that TEX12 expression could contribute to cancer, we examined the biology of TEX12 in tumourigenesis using mouse models of lymphoma and hepatocellular carcinoma (Fig. 6a–c). In an Eµ-myc model of MYC-driven B-cell lymphoma^39^, TEX12 protein was not detected in the mesenteric lymph nodes of naïve mice, but was aberrantly expressed in the lymph nodes of all lymphoma-positive mice, as assessed by immunohistochemistry (Fig. 6a). Further, high lymph node expression of TEX12 was correlated with a more aggressive disease and shortened survival (Fig. 6b); analogous observations were made in human ovarian and glioblastoma cancers (Suppl Fig. 1b, c). We also detected TEX12 expression in a diethylnitrosamine (DEN)-induced mouse model of hepatocellular carcinoma (HCC)^40,41^ (Fig. 6c), indicating that ectopic expression occurs in diverse tumour models. Importantly, TEX12 expression was detected as early as 5 weeks following DEN treatment, with peak levels observed at 40 weeks (Fig. 6c). Detection at the 5 week-stage indicates that TEX12 is expressed at the very early stages of oncogenesis, prior to the formation of an overt HCC tumour in mice. This observation highlights potential for TEX12 as an early diagnostic marker. In assessing how TEX12 expression might lead to poor prognosis in human cancers, we found that TEX12 expression and gene amplification each predict more aggressive disease (Suppl Fig. 1b, c).

Our experimental and bioinformatic analysis suggests that TEX12 is a potential driver of cancer progression. To test whether TEX12 is important for cancer cell growth, we performed siRNA knockdown of *TEX12* in multiple cancer cell lines (Fig. 6d and Supplementary Fig. 1d, e). In the majority of cell lines tested, *TEX12* silencing abrogated cellular proliferation, with a failure to increase cell numbers when cultured for a period of up to seven days (Fig. 6d). In contrast, TEX12-negative PEO1 cancer cells and non-transformed fibroblasts were not affected by *TEX12* silencing (Fig. 6d), ruling out off-target effects of siRNA treatment. We further confirmed that this proliferative defect was not simply due to cell death but due to a G2/M cell cycle arrest (Supplementary Fig. 1f, g), establishing that TEX12 is required for cell proliferation in a subset of human cancers. These findings suggest a pro-oncogenic function of TEX12 in a subgroup of cancers.

### Ectopic expression of TEX12 is regulated by retinoic acid signalling

**Fig. 7:**
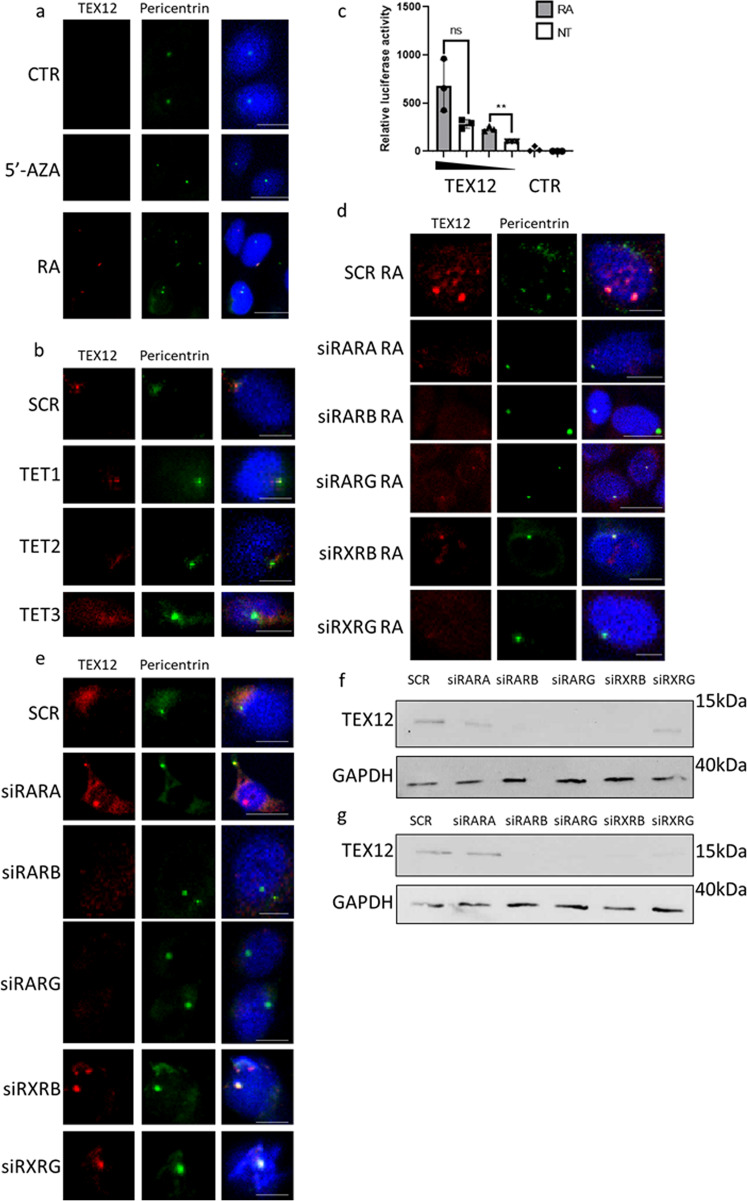
TEX12 re-expression is not regulated by demethylation but by retinoic acid signalling. **a** U2OS cells were left untreated or treated with 3 µM 5-azacytidine (5-AZA) or 1 µM retinoic acid. Bar = 20 µm. **b** PC3 cells were treated with siRNA as indicated. Bar = 20 µm. **c** HEK293T cells untransfected (CTR) or transfected with the increasing quantity of pGL4-TEX12 (221 bp) (as indicated by the triangle) or pGL4-SCR plasmid. Luciferase (Luc) activities were normalised to 1 µg plasmid NT. All data is shown as mean of normalised luciferase activities ± SD of three independent experiments and significant differences between each group were determined by student’s paired *t*-test (**p < 0.01, ns = not significant). **d** U2OS cells treated with or 1 µM retinoic acid and siRNA as indicated with corresponding quantification. NT non treated. TEX12 intensity measured in 10 cells per condition from three independent experiments. Bar = 20 µm. **e** PC3 cells treated with siRNA as indicated. Bar = 20 µm. **f** U2OS cells treated with or 1 µM retinoic acid and siRNA as indicated with corresponding quantification. **g** PC3 cells treated with siRNA as indicated.

Aberrant expression of TEX12 in somatic cells contributes to increased proliferation and centrosomal aberrations, both common features of cancer (Figs. 3 and 6). Previously, it has been proposed that aberrant expression of meiotic X-chromosome genes in cancer is driven by genome-wide DNA demethylation^42^. To investigate if the same mechanism is true for *TEX12* expression, TEX12-negative U2OS osteosarcoma cells were subject to chemical demethylation using 3 µM 5-azacytydine for 48 h^43,44^. These experiments showed that demethylation alone was not able to activate ectopic *TEX12* expression (Fig. 7a). Reciprocally, silencing the ten eleven translocation (TET1-3) demethylating enzymes for 72 h in TEX12-positive PC3 prostate cancer cells was not sufficient to suppress *TEX12* expression (Fig. 7b). However, 48 h of treatment with retinoic acid, a key activator of the meiotic programme^45^, successfully activated *TEX12* expression in somatic cells (Fig. 7a). We used bioinformatics to predict the *TEX12* promoter region of chr11: 112,167,162-112,167,382, which was cloned into a luciferase-reporter vector. A luciferase reporter assay confirmed that this region contains a promoter element that is activated by retinoic acid treatment (Fig. 7c). Finally, we assessed which retinoic acid receptors could play a role in *TEX12* expression. Whilst silencing of any retinoic acid receptor decreased retinoic-acid induced TEX12 protein levels, TEX12 was strongly diminished only upon silencing of the retinoic acid receptor B (*RARB*) or retinoid X receptor G (*RXRG*) (Fig. 7d). This observation was confirmed in PC3 cells, in which RARB was similarly found to be critical for TEX12 protein expression (Fig. 7e). Upstream of *TEX12* there are seven RARE sequences including a DR10 supporting the role of RARs in *TEX12* expression. Our data demonstrate that ectopic expression of TEX12 in somatic cells is activated by retinoic acid signalling (Fig. 7c) and implicate RARB in this process as it was the only receptor which played a role in both cell line models tested (Fig. 7d–g).

## Discussion

Increasing evidence implicates mis-expression of meiotic genes as one of the causes of oncogenic progression, likely through the promotion of genetic instability and disturbance of genome maintenance pathways in cancer cells^46–50^. Here we report that meiotic synaptonemal complex protein TEX12 is frequently mis-expressed in cancer. We also show that TEX12 has a previously overlooked localisation to the microtubule organising centres, in both meiosis and mitosis. Our data suggests that at these locations, TEX12 might promote stabilisation of pericentrin and centrosome amplification, which is driven by phosphorylation of a single conserved tyrosine.

What drives *TEX12* re-expression in cancer? It is possible that re-expression of X-chromosome located meiotic genes in cancer is regulated primarily by demethylation similarly to X-chromosome reactivation during early development. This has been previously proposed for *MAGE-1*^42,44,51^, and the *SSX* gene family^52^. However, epigenetic changes between autosomes and sex-chromosomes are regulated differently during spermatogenesis^53^ where autosomal genes often require deposition of bivalent epigenetic marks for transcriptional silencing post-meiosis^53^. We propose that divergent regulation of expression between X and autosomal genes could hold true in oncogenic re-expression. It has been previously reported that demethylation was unable to consistently induce expression of chromosome 6 located *RAGE-1*^43^. Similarly, we observed that demethylation alone was not the driver mechanism of *TEX12* re-expression; however, expression was activated via retinoic acid signalling. Furthermore, induction of *TEX12* re-expression following retinoic acid stimulation is of therapeutic relevance as retinoid treatment is used in oncology^54,55^ to force cancer cell differentiation, thereby reducing their stemness^56^. Our results suggest that this therapeutic approach should be closely evaluated, as long-term doses of RA may cause ectopic expression of meiotic proteins, such as TEX12, potentially contributing to centrosome amplification and other cellular aberrations.

TEX12 is an essential structural component of the central element of the synaptonemal complex, which mediates chromosome synapsis in meiosis^27^. Here, we report a localisation for TEX12, independent of the SC, within centrosomes of meiotic and cancer cells. During meiosis, SC assembly is initiated at recombination sites and facilitated by telomere-led rapid chromosomal movements, mediated by microtubules associated with the nuclear envelope, and by the bouquet configuration, in which telomeres cluster and are constrained near the microtubule organizing center (MTOC) in mouse spermatocytes^57^. This dramatic chromosome choreography suggests a requirement for close functional integration between the SC and MTOCs. Given that both the SC and MTOC are coiled-coil rich, a structural connection between the SC and MTOCs through a shared coiled-coil protein constituent, such as TEX12, may underlie their close functional relationship. TEX12 localisation to centrosomes has similarities with previous observations in which SC components were found to localise to MTOCs/spindle-pole bodies, including 6C6 in plants^58^, and Sme4 in Sordariales^59,60^. However, this is the first report of centrosomal/MTOC localisation of a mammalian SC component. Thus, our findings suggest a possibility for an evolutionarily conserved cross-talk between structural components of the mammalian SC and MTOCs.

## Materials and methods

### Antibodies and plasmids

The anti-TEX12 (ab122455), anti-pericentrin (ab28144), anti-gamma tubulin (ab191114), anti-centrin 1 and 2 (ab11257) and anti-SYCE2 (ab107745) antibodies were obtained from Abcam and anti-FLAG (F1804) antibody was purchased from Sigma. Human TEX12 sequences, corresponding to amino acids 1–123 (full length) and 49–123 (core) and 45–123, 1–113, 1–91, 1–56 and 87–123, and SYCE2 were cloned into the pCMV-HA vector (Addgene Catalogue no 631604) for mammalian expression with an N-terminal FLAG-tag.

### Cell culture, transfections and lentiviral transductions

LNCaP and PC3 prostate cancer, MCF7 and MX1 breast cancer, WM1361 NRAS mutant and WM164 BRAF mutant melanoma, 3T3-L1 mouse fibroblasts and COS-7 cells were obtained from American Type Culture Collection (Manassas, USA). LNCaP, PC3 and MX-1 cells were maintained in RPMI 1640 media and remaining cell lines in DMEM media supplemented with 2 mM l-glutamine (Invitrogen) and 10% (v/v) fetal calf serum (FCS) at 37 °C in 5% CO_2_. Cell lines were never maintained for more than 30 passages or 2 months of continuous culturing. Cell lines were tested for mycoplasma on a tri-monthly basis. Proliferation was measured by live cell imaging with the Incucyte system every 6 h for 156 h post-treatment. Transfections were performed using Lipofectamine2000 reagent (Invitrogen) following the manufacturer’s instructions.

### siRNA gene silencing and gene expression analysis

The *TEX12* targeting siRNA sequences were (A) AAGCCUUGGAGAAAGAUUUAAAU[dTdT], (B) CAGCAGUAGAUGCAUCUUACA[dTdT] and (C) SASI_Hs01_00169803 (Sigma). Cells were reverse transfected with siRNA using RNAiMax (Invitrogen) according to manufacturer’s instructions and incubated in culture media for 96 h prior to cell lysis and analysis. For real-time qPCR, total RNA was extracted using TRIzol (Invitrogen, 15596-026), RNA quality and yields were assessed using a NanoDrop 2000 (NanoDrop), 1 μg of total RNA was reverse transcribed using SuperScript VILO (Invitrogen, 11755-050) and qPCR performed using QuantiTect SYBR Green (QIAGEN, 204143) on an ABI PRISM 7500 Sequence Detection System (Applied Biosystems). Data was tested for parametric distribution. Parametric data was analysed using appropriate *t*-tests or ANOVA with Bonferroni’s comparison test for multiple group comparisons. Non-parametric data was analysed using Wilcoxon signed-rank test. By convention, ***p-values < 0.001, **p-values < 0.01 and *p-values < 0.05.

### Immunohistochemistry

Antigens were retrieved by microwaving the slides in 10 mM citrate pH 6.0 for 15 min followed by staining the tissues. Antibodies were detected with ImmPRESS HRP IgG (Vector labs). Samples were scored blind using the histoscore methodology^61,62^. Briefly, percentage and intensity of staining for positive cells was estimated (0, 1, 2, 3) using the following equation: *H*-score = (% of cells with low level positivity) + 2 × (% of cells with medium level positivity) + 3 × (% of cells with high level positivity). For survival analysis, high marker levels were defined as a value in the third and fourth upper quarter of the population.

### Immunofluorescence

For Immunofluorescence, cells were fixed for 10 min in ice cold methanol followed by blocking in 1% BSA, staining for 1 h at room temperature in primary antibodies then for 1 h at room temperature with goat anti-rabbit Alexa Flour594 (A32740) and rabbit anti-mouse Alexa Fluor488 (A-11059) Life Technologies secondary antibodies at 1:250 dilution and DAPI, mounting in prolong antifade gold reagent (Life Technologies P36961) and imaging using LSM 780 confocal microscope and processed with Zen 2.6 blue edition software. For measuring staining intensity the centrosome area was marked out and the average pericentrin fluorescence intensity was measured within that area as previously described^63^.

### Flow cytometry

Cell cycle stage assessment cells were fixed in 70% ethanol and frozen at −20 °C for at least 24 h prior to staining and flow cytometry which were performed using Cell Cycle analysis Kit from Guava (Millipore, 4500-0220) and analysed in a Guava® easycyte HT (Millipore) cytometer as per manufacturers instructions.

### Mouse models

In order to induce liver cancer, day old 15 wild-type mice were given 30 mg/kg DEN in 0.9% saline by IP injection. Cohorts of mice were humanely killed, and liver tissue was harvested at various time-points (5 weeks, 20 weeks, 30 weeks and 40 weeks) post DEN administration. Eμ-Myc mice^39^ were purchased from The Jackson Laboratory, Maine, USA and wild-type (C57Bl/6) were purchased from Charles River, UK. Colonies were established and maintained in the Comparative Biology Centre, Newcastle University, according to the FELASA Guidelines. No blinding of groups in mouse studies was performed. All mice were designated to an experimental group dependent on their genotype. To perform survival analysis, Eμ-Myc transgenic male mice were monitored daily and were sacrificed at pre-determined end-points, defined as the animal becoming moribund, losing bodyweight/condition and/or having palpable tumour burden at any lymphoid organ site. At this point, mice were necropsied and samples taken for downstream analysis.

All animal experiments were approved by Newcastle University’s Animal Welfare and Ethical Review Board. All procedures, including the breeding of genetically modified, Eμ -myc mice, were carried out under project and personal licences approved by the Secretary of State for the Home Office, under the United Kingdom’s 1986 Animal (Scientific Procedures). All breeding was undertaken according to FELASA guidelines.

### Spermatocyte staining

Testis were dissected from freshly sacrificed mice and processed for cell squashes as previously described. This project was conducted in accordance with the ILAR Guide for the Care and Use of Laboratory Animals and the UC Davis Animal Welfare Assurance on file with the US Public Health Service, under the approval and surveillance of the UC Davis Institutional Animal Care Committee. Briefly, seminiferous tubules were fixed in freshly prepared 2% formaldehyde containing 0.1% Triton-X for 10 mins at room temperature. Small pieces of tubules were placed on a glass slide, minced gently and then squashed under a coverslip with pressure from the blunt end of pencil. Slides were then frozen briefly in liquid nitrogen and coverslips removed. Following three washes with PBS, immunofluorescent staining was performed as described, using the following primary antibodies with incubation overnight at room temperature: rabbit anti-TEX12 (Abcam, ab122455;1:200 dilution), mouse anti-Centrin 1 (Millipore, 04-1624; 1:200 dilution), mouse anti-SYCP3-488 (Abcam, ab205846; 1:100 dilution), mouse anti-γ-Tubulin-647 (Abcam, ab191114; 1:100 dilution). Goat secondary antibodies were then added for 1 h at 37° C, either anti-rabbit 488 (Life Technologies, 31822; 1:1000 dilution), or anti-mouse 594 (Life Technologies, A-11032, 1:1000 dilution). Finally, slides were rinsed, stained with DAPI and mounted with ProLong Gold antifade reagent. Images were acquired using a Zeiss AxioPlan II microscope, ×63, 1.4 NA objective and X-Cite light source. Images were captured by a Hamamatsu ORCA-ER CCD camera and processed using Volocity (PerkinElmer).

### Recombinant protein expression and purification

Human TEX12 sequences, corresponding to amino acids 1–123 (full length) and 49–123 (core), were cloned into the pMAT11 vector^64^ for bacterial expression as a fusion protein to an N-terminal His_6_-MBP-tag, separated by a tobacco etch virus (TEV) site for tag cleavage. Proteins were expressed in BL21(DE3) *E. coli* cells (Novagen®), cultured in 2xYT media and induced with 0.5 mM IPTG at 25° for 16 h. Cells were lysed in 20 mM Tris pH 8.0, 500 mM KCl, by sonication and clarified lysate was applied to Ni-NTA resin (Qiagen) followed by amylose resin (NEB). Proteins were further purified by TEV protease cleavage for tag removal, anion exchange (GE Healthcare) and stored at −80 °C in 20 mM Tris pH 8.0, 150 mM KCl, 2 mM DTT after concentration (PALL Microsep™ Advance, 3kD) to 6-12 mg/ml. Proteins were analysed by SDS-PAGE (NuPAGE Bis-Tris system) with SimplyBlue SafeStain (Invitrogen). UV spectrophotometry (Cary 60 UV-Vis, Agilent Technologies) was used to calculate protein concentrations with theoretical molecular weights and extinction coefficients determined by ExPASY ProtParam.

### Circular dichroism (CD) spectroscopy

CD data were collected using a Jasco J-810 spectropolarimeter (Institute for Cell and Molecular Biosciences, Newcastle University). CD spectra for TEX12 and TEX12_Core_ were collected in 10 mM NaH_2_PO_4_ pH 7.5, 150 mM NaF, at 0.36 and 0.24 mg/ml, respectively. Measurements were recorded at 4 °C between 260 and 185 nm, with 0.2 nm intervals, in a 0.2 mm pathlength quartz cuvette (Hellma). Nine measurements were collected and averaged, with buffer correction, and converted to mean residue ellipticity (MRE [θ]). The DichroWeb server (http://dichroweb.cryst.bbk.ac.uk) was used for used for deconvolution with the CDSSTR algorithm. CD thermal melts were recorded in 20 mM Tris pH 8.0, 150 mM KCl, 2 mM DTT, at 222 nm with a temperature range of 5-95 °C and a 1 °C per minute ramping rate. Data were plotted as % unfolded after conversion to MRE ([θ]_222,x_-[θ]_222,5_)/([θ]_222,95_-[θ]_222,5_), with melting temperatures (Tm) determined by the temperature at which the sample is 50 % unfolded.

### Size-exclusion chromatography multi-angle light scattering (SEC-MALS)

SEC-MALS was performed to determine the absolute molecular masses of TEX12 and TEX12_Core_. Protein samples, at 12 and 4 mg/ml, respectively, were loaded onto a pre-equilibrated (20 mM Tris pH 8.0, 150 mM KCl, 2 mM DTT) Superdex™ 200 Increase 10/300 GL SEC column (GE Healthcare) with an ÄKTA™ Pure (GE Healthcare) at 0.5 ml/min. The column flow-through was directed to a DAWN HELEOS II MALS detector (Wyatt Technology) and then to an Optilab T-rEX (Wyatt Technology) differential refractometer. ASTRA® 6 software (Wyatt Technology) was used calculate molecular weights from the eluted peaks, by Zimm plot extrapolation using a 0.1850 ml/g dn/dc value.

### Size-exclusion chromatography small-angle X-ray scattering (SEC-SAXS)

SEC-SAXS data collection was carried out at the B21 beamline at the Diamond Light Source synchrotron facility (Oxfordshire, UK). TEX12 and TEX12_Core_, at 12 and 6 mg/ml, respectively, were loaded onto a pre-equilibrated (20 mM Tris pH 8.0, 150 mM KCl, 2 mM DTT) Superdex™ 200 Increase 10/300 GL SEC column (GE Healthcare) with an Agilent 1200 HPLC system at 0.5 ml/min. The column flow-through was directed to the experimental cell for SAXS data collection in 3 s frames, with a 4.014 m detector distance at 12.4 keV. ScÅtter 3.0 (http://www.bioisis.net) was used to analyse SAXS data. Data were subtracted for buffer, and then averaged for Guinier (Rg) and cross-sectional radius (Rc) analysis. *P(r)* distributions were generated using PRIMUS^65^.

### Statistics and reproducibility

All data was first tested for parametric distribution and analysed with GraphPad PRISM software. Parametric data was analysed using appropriate *t*-tests or ANOVA with Bonferroni’s comparison test for multiple group comparisons. Non-parametric data was analysed using Mann–Whitney or Wilcoxon signed-rank test. By convention, ***p-values < 0.001, **p-values < 0.01, and *p-values < 0.05. Number of experiments is indicated for each figure where experiments with internal replicates are shown ±SEM while experiments were that was not possible, e.g. animal work, are shown ±SD.

### Reporting summary

Further information on research design is available in the Nature Research Reporting Summary linked to this article.

## Supplementary information

Supplementary Information

Description of Additional Supplementary Files

Supplementary Data 1

Reporting Summary

## Data Availability

All data and source data and raw blots has been made available in supplementary Fig. 4 and Supplementary data 1 files. All other information are available from the corresponding author on reasonable request.
