# Supplementary material for: Centrosome dysfunction associated with somatic expression of the synaptonemal complex protein TEX12
Source: Commun Biol. 2021 Dec 8;4:1371. doi: 10.1038/s42003-021-02887-4 (PMC8654964; doi:10.1038/s42003-021-02887-4)
Supplement: Supplementary file 1 — Supplementary Information [file 42003_2021_2887_MOESM1_ESM.pdf]

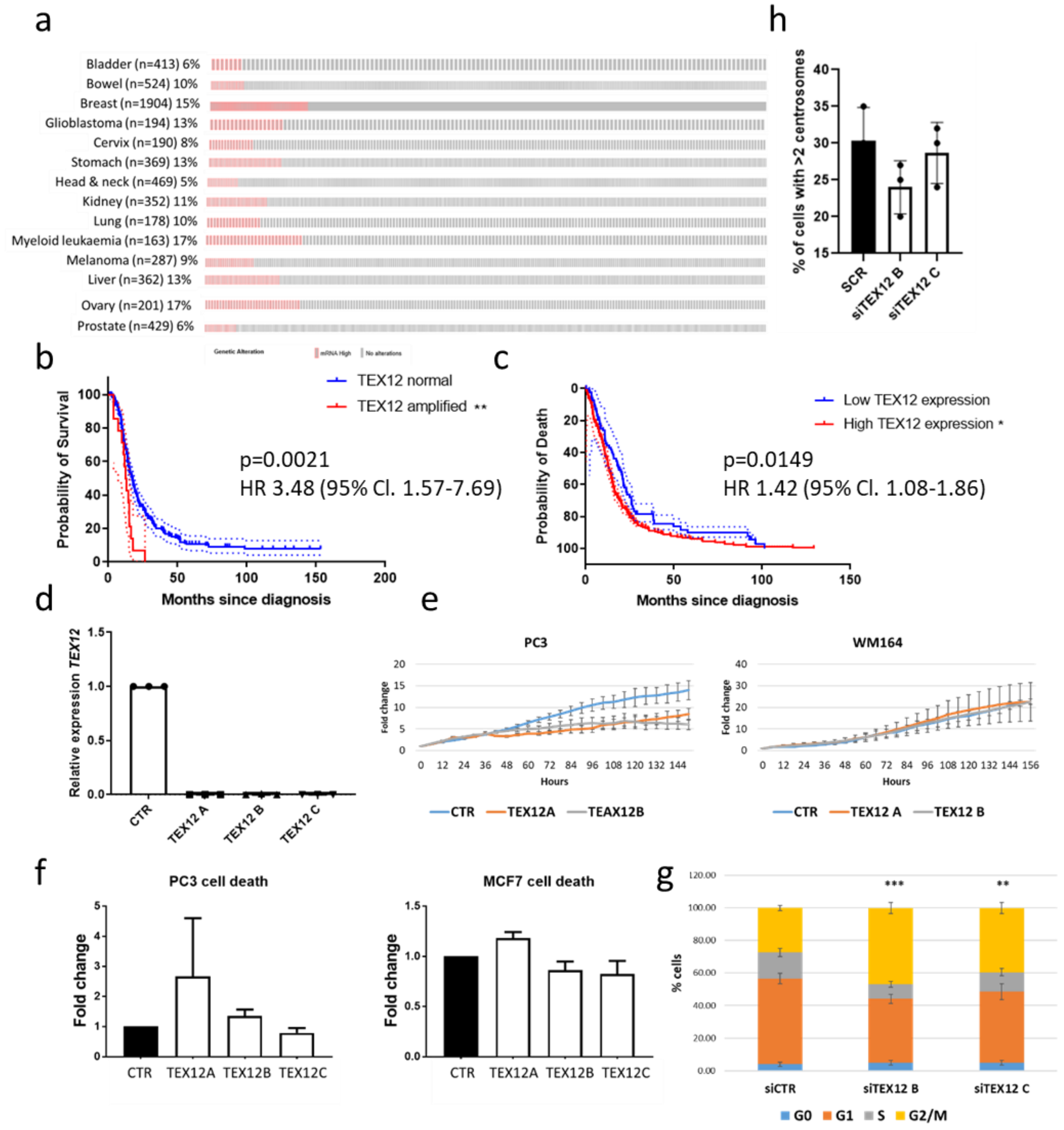

<u>*Supplementary Figure 1 TEX12 is marker of poor prognosis critical for cancer cell proliferation.*</u> *(**a**) TCGA dataset analysis demonstrating frequency of TEX12 re-expression at the mRNA level. (**b**) Kaplan-Meier curve of TCGA dataset of ovarian cancer (n=261). Patients were divided based on presence and absence of TEX12 amplification. * indicates p<0.05 compared to TEX12 normal patients. 95% confidence intervals are indicated with dotted*

*lines. (**c**) Kaplan-Meier survival curve of TCGA dataset of glioblastoma (n=349). Patients were divided based on high versus low TEX12 transcripts. * indicates p<0.05 compared to TEX12 low expressing patients. 95% confidence intervals are indicated with dotted lines. (**d**) MX1 cancer cells were treated with siRNA as indicated for 96 hours and after RNA extraction TEX12 transcript levels were measured with quantitative RT-PCR and normalised to HPRT1 transcript levels. Data represents three independent experiments +/- SEM. (**e**) Cells were treated with siRNA and cell growth was measured every 3h with IncuCyte. Data represents three independent experiments +/- SEM. (**f**) Cells were treated with siRNA for 156h and cell death was measured with CellTox. Data represents three independent experiments +/- SEM. (**g**) % of PC3 cells in different phases of cell cycle after 72hrs scrambled (CTR) or TEX12 targeting siRNA treatment measured with Guava. Data represents three independent experiments +/- SEM. *** indicates p<0.001 as assessed by T-Test. (**h**) % of MCF7 cells with amplified centrosomes was established 72 hours post siRNA treatment with scrambled (SCR) or TEX12 targeting siRNAs. Data represents three independent experiments +/- SD.*

*Supplementary Figure 2 Antibody validation. (**a**) COS7 cells were transfected with empty vector control plasmid (EV) or TEX12-FLAG for 48h followed by immunofluorescence with TEX12 (ab122455) and Pericentrin antibodies. (**b**) MX-1 cells were treated with control siRNA (CTR) or siTEX12 B for 96h followed by immunofluorescence with TEX12* (ab122455) *antibody. (**c**) COS7 cells were transfected with empty vector control or TEX12-FLAG for 96h followed by cell lysis and Western blotting with TEX12 (ab122455) antibody. (**d**) COS7 cells were transfected with empty vector control plasmid (EV) or TEX12-FLAG for 48h followed by immunofluorescence with TEX12 (ab122455) and FLAG M2 antibodies.*

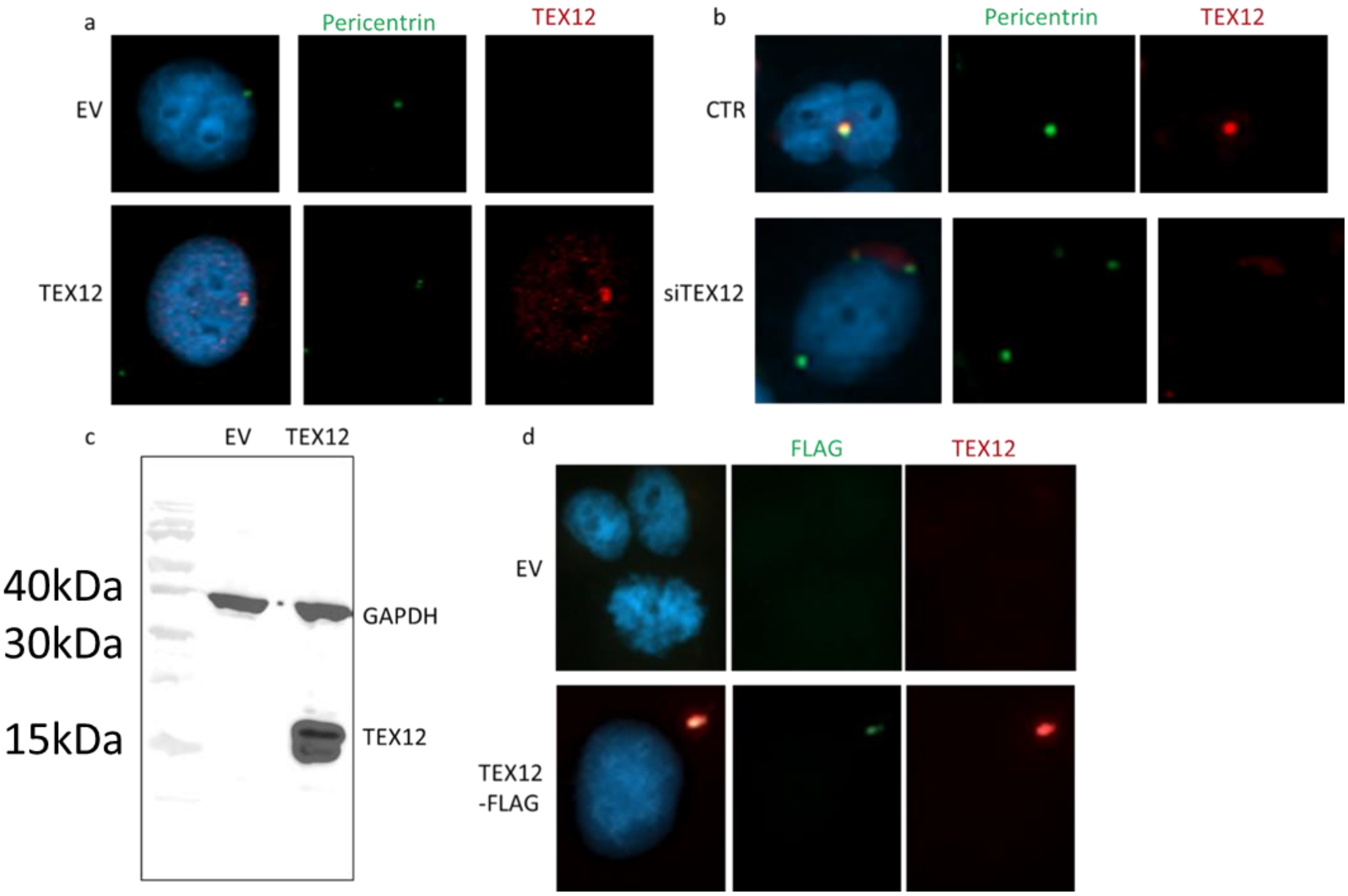

*<u>Supplementary Figure 2 Antibody validation.</u> (**a**) COS7 cells were transfected with empty vector control plasmid (EV) or TEX12-FLAG for 48h followed by immunofluorescence with TEX12 (ab122455) and Pericentrin antibodies. (**b**) MX-1 cells were treated with control siRNA (CTR) or siTEX12 B for 96h followed by immunofluorescence with TEX12* (ab122455) *antibody. (**c**) COS7 cells were transfected with empty vector control or TEX12-FLAG for 96h followed by cell lysis and Western blotting with TEX12 (ab122455) antibody. (**d**) COS7 cells were transfected with empty vector control plasmid (EV) or TEX12-FLAG for 48h followed by immunofluorescence with TEX12 (ab122455) and FLAG M2 antibodies.*

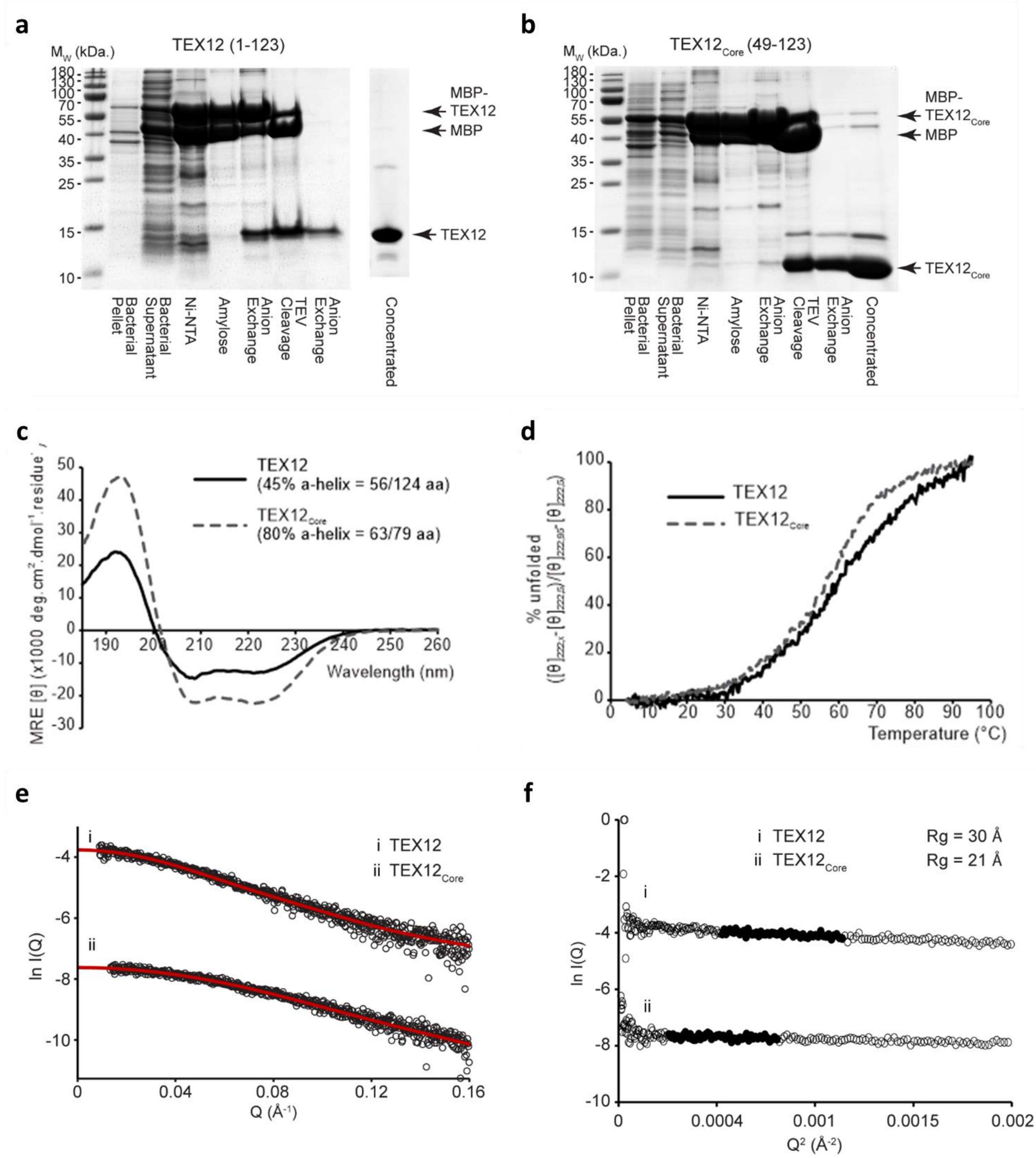

<u>*Supplementary Figure 3 Purification and SAXS analysis of recombinant TEX12.*</u> *(**a-b**) SDS-PAGE of recombinant expression and purification of (**a**) TEX12 (amino acids 1-123) and (**b**) $TEX12_{Core}$ (amino acids 49-123) through Ni-NTA, amylose, and anion exchange chromatography, following by TEV cleavage to remove N-terminal MBP tags, with subsequent anion exchange and concentration. (**c**) Far UV CD spectra and (**d**) CD thermal denaturation of TEX12 (solid line) and $TEX12_{core}$ (dashed line). (**c**) Secondary structure composition was estimated through deconvolution of spectra with data fitted*

at normalised r.m.s. deviation values of 0.015 and 0.008, respectively. (***d***) Thermal denaturation was recorded as % unfolded based on the helical signal at 222 nm; melting temperatures were estimated at 60°C and 57°C, respectively. (***e-f***) SEC-SAXS analysis of TEX12 and TEX12$_{core}$. (***e***) Scattering intensity plots; experimental data are shown in open circles with P(r) distribution fits displayed as red lines. (***f***) Guinier analysis to determine the radius of gyration (Rg) of TEX12 and TEX12$_{core}$ with linear fits shown in black. Q.Rg values were < 1.3.

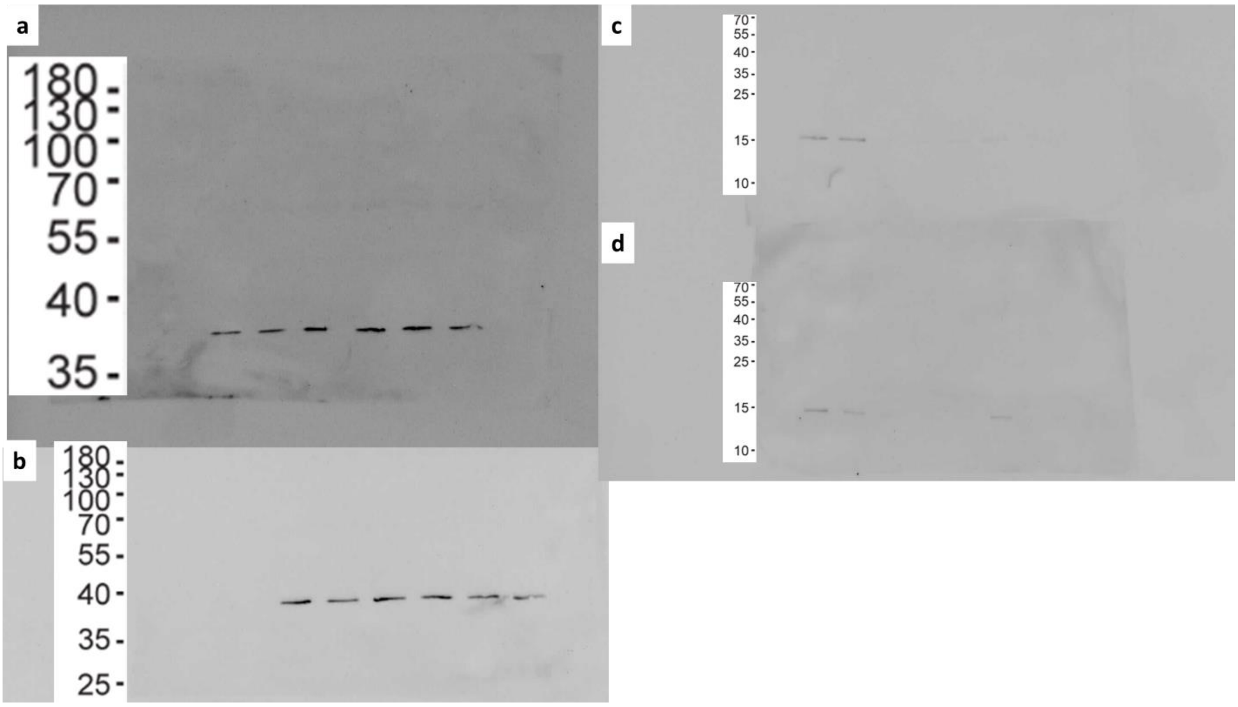

Supplementary Figure 4 Full blot images from Figure 7. *(**a**) represents Figure 7f GAPDH. (**b**) represents Figure 7e GAPDH. (**c**) represents Figure 7f TEX12 and (**d**) represents Figure 7e TEX12.*
